# Ranges of control in the transcriptional regulation of Escherichia coli

**DOI:** 10.1186/1752-0509-3-119

**Published:** 2009-12-24

**Authors:** Nikolaus Sonnenschein, Marc-Thorsten Hütt, Helga Stoyan, Dietrich Stoyan

**Affiliations:** 1 School of Engineering and Science, Jacobs University Bremen, Campus Ring 1, 28759 Bremen, Germany; 2 Institute of Statistics, TU Bergakademie Freiberg, Prüferstraße 9, 09596 Freiberg, Germany

## Abstract

**Background:**

The positioning of genes in the genome is an important evolutionary degree of freedom for organizing gene regulation. Statistical properties of these distributions have been studied particularly in relation to the transcriptional regulatory network. The systematics of gene-gene distances then become important sources of information on the control, which different biological mechanisms exert on gene expression.

**Results:**

Here we study a set of categories, which has to our knowledge not been analyzed before. We distinguish between genes that do not participate in the transcriptional regulatory network (i.e. that are according to current knowledge not producing transcription factors and do not possess binding sites for transcription factors in their regulatory region), and genes that via transcription factors either are regulated by or regulate other genes. We find that the two types of genes ("isolated" and "regulatory" genes) show a clear statistical repulsion and have different ranges of correlations. In particular we find that isolated genes have a preference for shorter intergenic distances.

**Conclusions:**

These findings support previous evidence from gene expression patterns for two distinct logical types of control, namely digital control (i.e. network-based control mediated by dedicated transcription factors) and analog control (i.e. control based on genome structure and mediated by neighborhood on the genome).

## Background

The circular genome of *E. coli *is still an object of intense scientific research (see, e.g., [1]). It is a rich source of information on the organization of gene regulation, the interplay of different types of control exerted on gene expression, a model system for analyzing DNA topology, the model for which the most detailed electronically accessible transcriptional regulatory network has been compiled.

Many processes, acting on a broad range of scales, contribute to the evolution of bacterial chromosomes. Genes are organized in operons, i.e., groups of genes sharing a regulatory domain. The genome is shaped by point mutations, large-scale rearrangements, strand breaks and inversions during replication. The gene inventory is modified by gene duplications or deletions and lateral or horizontal transfer of genes. It is striking that an ever closer look at statistical properties of data reveals ever more systematic information, shaped by evolution, on an ever broader range of length scales.

Starting from the work by De Martelaere and Van Gool (1981) [2] and Jurka and Savageau (1985) [3] the gene density along the circular chromosome of E. coli has been discussed as a potential source of information on the evolutionary shaping of the system and in particular as a means of using DNA topology (i.e. the 3D structure of the genome) for regulatory purposes (see also [4]).

The papers by Warren and ten Wolde (2004) [5] and Képès (2004) [6] focus on distances between genes or operons. Both are studies of the specific patterns in the distributions of distances between regulatory pairs (genes or operons regulating each other or pairs of genes or operons co-regulated by other genes). Warren and ten Wolde (2004) [5] find a substantially reduced distance between operons in such regulatory pairs, suggesting an evolutionary pressure to reduce such distances for efficient regulation. For obtaining this, they use classical characteristics of point process statistics, namely partial pair correlation functions and nearest neighbor distance probability density functions.

Képès (2004) [6] observe a periodicity in the distances between regulator and target, where the period length is in the same order of magnitude as known loop domains in the 3D organization of the *E. coli *chromosome.

**Figure 1:**
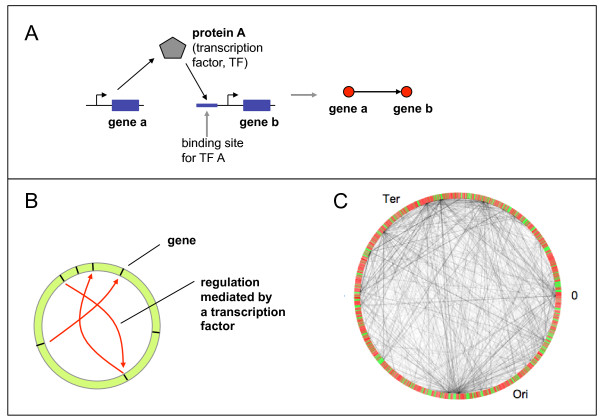
**Schematic view on the transcriptional regulatory network**. (A) For the TRN, the nodes are genes and the (directed) links describe the regulatory action of one gene onto another mediated by a transcription factor. More specifically, for the link shown in the Figure, gene *a *expresses protein *A*, which serves as a transcription factor (TF) binding in the regulatory region of gene *b *and thus controlling gene *b*. The links of the transcriptional regulatory network can be inserted into the circular genome of *E. coli *(schematically shown in (B) and for the real genome in (C), based on the data from RegulonDB, version 6.2.

More recently, Hermsen et al. (2008) [7] observed that genes with opposite orientation have a bias towards larger distances, when oriented away from each other (divergent gene pair; e.g. the second gene pair in Figure 1) compared to those oriented towards each other (convergent gene pair; e.g. the first gene pair in Figure 1). They argue that this bias is due to the larger size of the upstream control region compared to the downstream control region.

Darling et al. (2008) [8] discuss biases in genomic inversions with respect to the replichores and other patterns of genome rearrangement in bacterial chromosomes. Another important factor influencing gene-gene distance statistics on a very general level is gene clustering. The origin of observed gene clustering is attributed to gene duplication and divergence, an evolutionary advantage of clustering, as it might increase a gene's chance for horizontal gene transfer or, lastly, selective advantage of gene clusters due to functional coupling and the efficient organization of transcription (see the discussion in [9]). From the systems perspective, mainly the regulatory control mediated by direct binding of transcription factors has been investigated. The compilation of these interactions for *E. coli *into a database [10] allows the construction of a transcriptional regulatory network (TRN) [11]. This view yields deep topological insights into the hierarchical organization of TRNs (Ma et al., 2004 [12]; Yu and Gerstein, 2006 [13]) and their composition out of specific network motifs (Shen-Orr et al., 2002 [11]). The TRN has been used for the interpretation of expression patterns (Gutierrez-Rios et al., 2003 [14]; Herrgard et al., 2003 [15]), revealing both the potential and the limitations of this perspective. In particular, recently it became obvious that other effects with very different regulatory mechanism have to be taken into account, like alterations of the DNA structure on a small [16,17] and larger [18] scale. Thus, understanding the organizational logic of gene regulation necessitates a clear distinction of the different control types in the first place, as a prerequisite for the assessment of their impact in regulation.

Another link between these two research areas, gene distribution and TRN, comes from the observation that gene neighborhood explains some features of observed gene expression patterns (Marr et al. 2008 [19]; Blot et al. 2006 [20]). In particular, Marr et al. (2008) [19] analyze the interplay between two types of control in gene expression profiles in *E. coli*, one network-mediated and the other mediated by DNA topology.

These two control types have been termed *digital *(referring to the fact that the TRN provides static information on the connections between unique, discontinuous components, e.g. a particular pair of regulator and regulated gene) and *analog *(referring to the fact that the expression of specific genes is under the control of continuous information provided by distributions of supercoiling energy in the genome), respectively [19].

The statistical properties of gene distributions and gene spacings have been studied to detect deviations from randomness and interpret these deviations in a suitable evolutionary context. To a large extent, these investigations differ (apart from the technical details of the statistical tools and the construction of suitable null models) predominantly in the categories of genes analyzed. In the present paper we show results for two analysis steps, where the first analysis distinguishes between two classes. Analysis I discusses genes involved in regulation (i.e., either being regulated by a transcription factor or producing a transcription factor regulating other genes; class 1) and genes not involved in regulation mediated by transcription factors (which in the following we will call "isolated genes"; class 2). Analysis II consists of pairs of genes regulated by a common transcription factor. Distances between the genes in such a pair will be contrasted to the distances between arbitrary genes.

The biological hypothesis behind these categories is that different means of gene regulation essentially have different length scales. The novel feature of our approach lies in two points: (1) the distribution of regulated/regulating genes vs. (regulatorily) isolated genes has not been studied before. Our finding here, a pronounced deviation from randomness for the isolated genes, fits to the hypothesis stemming from previous investigations of control types in gene expression patterns (Marr et al. 2008 [19]); (2) in order to detect deviations from randomness we employ different non-classical types of correlation functions. Our hypothesis, based on the findings from Marr et al. (2008) [19], is that the existence of distinct logical types of control (namely digital and analog) has a systematic impact on the statistical features of gene distributions. In particular, distances between isolated genes and all others should be smaller than average distances between genes, as isolated genes tend to be co-regulated by spatial neighborhood via the 3D structure of the genome.

Results are in the following presented both on the level of individual genes and on the level of operons.

## Results and Discussion

First we present the gene distance distributions for the two gene classes, (isolated genes and genes involved in regulation; see above). Then we discuss pair correlation functions *g*(*s*), partial pair correlation functions *g*_*ij*_(*s*), mark connection functions *p*_*ij*_(*s*), connectivity correlation functions *c*(*s*), and control correlation functions *k*_3_(*s*) (see Materials and Methods).

**Figure 2:**
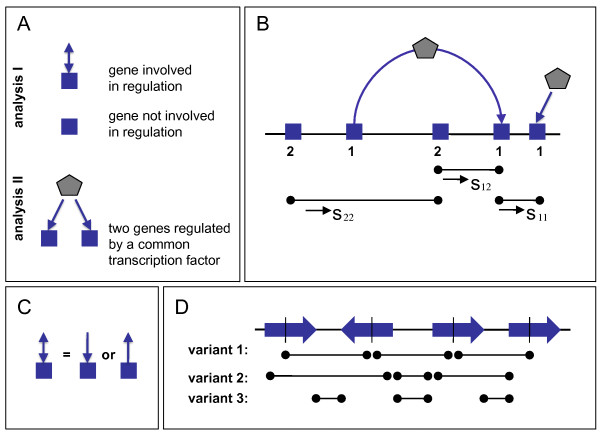
**Definitions of categories and distances**. (A) The classes of genes (involved and not involved in regulation) entering Analysis I and the subset of genes involved in regulation (pairs of genes under common regulation), which is studied in Analysis II. (B) Examples of distances *s*_11_, *s*_12 _and *s*_22 _entering point process analysis. (C) No distinction is made between genes receiving a regulatory influence and genes encoding transcription factors regulating other genes. (D) Various possibilities of defining the distance of two genes along the genome. In this investigation we use variant 1.

Figure 2 explains the categories of genes (operons) we are studying. In the first part (Analysis I; classes 1 and 2; cf. Figure 2A and 2C) we are looking at statistical properties of shortest distances between two genes involved in regulation (*s*_11_), two isolated genes (*s*_22_), and an isolated gene, together with a gene involved in regulation (*s*_12_); cf. Figure 2B. In the second part (Analysis II) we study distances between two genes regulated by a common transcription factor. In all cases we analyze the shortest distance (in base pairs, bp) along the circular genome to the nearest neighbor of the respective type. We do not consider the orientation of genes on the genome or the sizes of the genes and operons. In fact, we represent every gene only by a single point, namely by its center. We checked that our results do not change qualitatively when we consider other definitions of the "distance" between two genes (e.g., from start points to end points or the minimal distance between any two points of the two genes; cf. Figure 2D). We ignore biases induced by the relative position of the gene under consideration with respect to the origin of replication (ori) or the Ter macrodomain, respectively.

Thus we are confronted with problems of point process statistics (see Materials and Methods), where the genes or operons are the points. They are marked by 1 or 2, corresponding to the classes above, 1: involved in regulation, 2: isolated.

**Figure 3:**
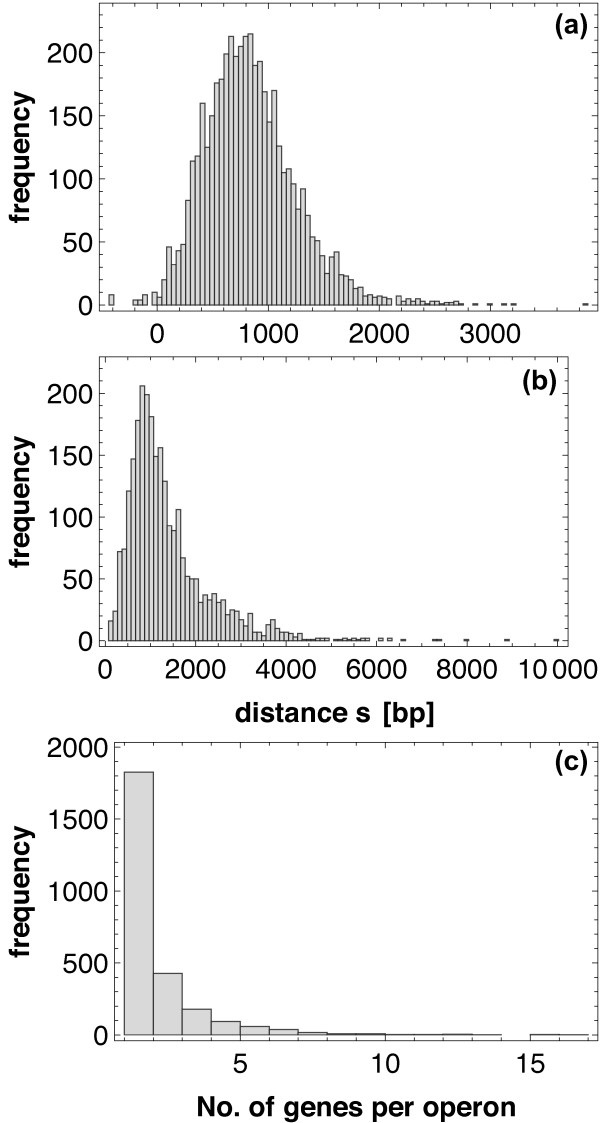
**Pairwise distances and operon sizes**. Histogram of pairwise distances for (a) genes and (b) operons, and distribution of operon sizes (c).

Figure 3 shows the distributions of shortest distances on the gene level (Figure 3a) and on the operon level (Figure 3b), together with the distribution of gene content of operons (Figure 3c; i.e. the number of genes in an operon). Most of the operons in *E. coli *consist of only a single gene, some operons, however, contain as many as 15 genes. Figure 3 already reveals several interesting features of the data: Distances between operons tend to be larger than distances between genes (which results from the systematic omission of intra-operon distances, when passing from genes to operons); the decrease in frequency with the distance does not seem to follow an exponential distribution, suggesting a deviation from a Poisson process (and therefore from a random distribution of points).

**Figure 4:**
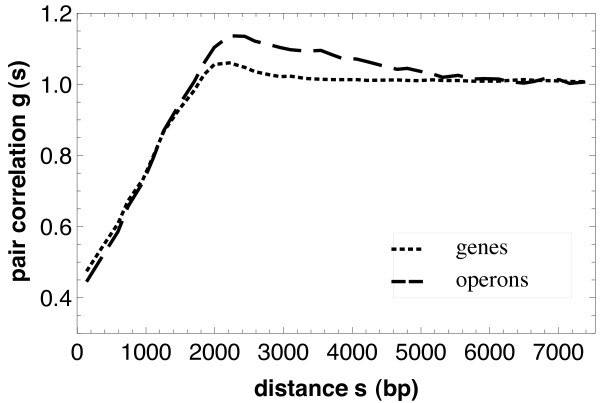
**Pair correlation functions**. The pair correlation function *g*(*s*) for genes (dotted) and operons (dashed). The functions indicate a weak tendency of regularity of the gene/operon positions.

After the discussion of the nearest neighbor distances we report now on the correlation functions. First we discuss the pair correlation functions *g*(*s*), see Figure 4. They indicate the well-known fact that the positions both of the genes and operons are not completely randomly distributed, i. e. according to a Poisson process, where *g*(*s*) = 1. In contrast, they are more regularly distributed, probably simply due to the finite size of the objects, as is shown by the values of *g*(*s*) smaller than 1 for small *s*.

Typical gene sizes range from a few hundred bp to several thousand bp with the mean size centered around 1 kpb.

There is even a maximum for distances of 1 and 2 kbp, while for larger values the curves approach fast the value 1, which corresponds to absence of location correlation. The range of correlation is for the operons somewhat longer than for the genes, it goes until 6 kbp.

A suitable tool for analyzing the relative contributions of the different categories to these correlations is the partial *pair correlation function g*_*ij*_(*s*) with *ij *= 11 (between genes involved in regulation), *ij *= 22 (between isolated genes) and *ij *= 12 (one gene involved in regulation, the other isolated), respectively.

**Figure 5:**
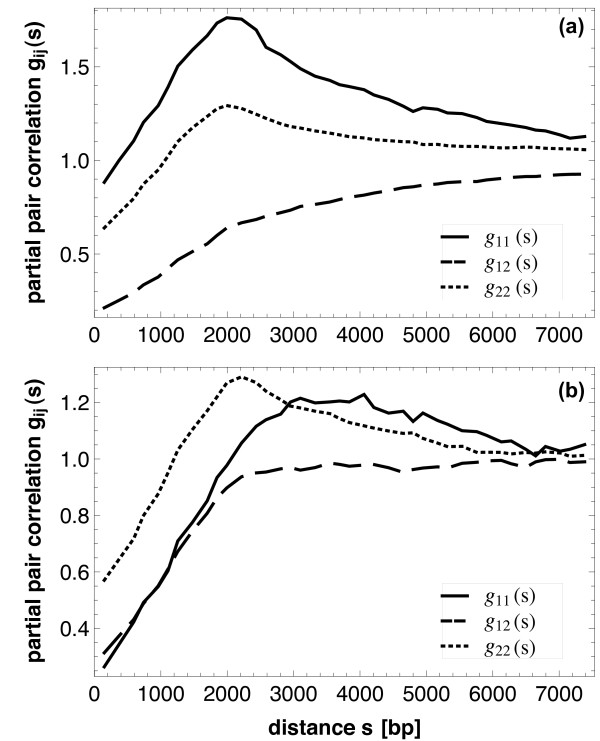
**Partial pair correlation functions**. Partial pair correlation functions *g*_*ij*_(*s*) for (a) genes and (b) operons. In both cases the full curve denotes *g*_11_(*s*), the dotted curve *g*_22_(*s*) and the dashed curve *g*_12_(*s*). The functions indicate a weak tendency of regularity of isolated and regulated points, and a clear tendency of repulsion between isolated and regulated points.

Figure 5 shows the curves *g*_*ij*_(*s*) for genes (Figure 5a) and for operons (Figure 5b). The results for *g*_11 _and *g*_22 _are similar to those from Warren and ten Wolde (2004) [5] and, in fact, display similar features as the *pair correlation function g*(*s*) in Figure 4: very small distances are suppressed (due to the finite size of the elements); one observes a peak between 1 and 2 kbp and then a convergence to the value 1 as for the uncorrelated case. The ranges of correlation are between 5 kbp and 7 kbp.

The curves for *g*_12_(*s*), however, are new and, to a certain extent, unexpected: the distances between isolated and regulatory genes do not show a peak at intermediate distances. Obviously, the repulsion between isolated and regulatory genes is stronger and longer than that of genes of the same type, namely 7 kbp. In contrast, for operons it is shorter, only 3 kbp.

The term "repulsion" is used here in a simplifying sense, in order to say that there is a tendency that the distances between isolated and regulatory genes are larger than between genes of the same type. This may be a result of real repulsion as well as of relative "attraction" of the members of one class towards itself. We interpret this repulsion as an unmixing of genes predominantly regulated by transcription factors (digital control; cf. [19]) and genes predominantly regulated by the 3D structure of the genome (analog control). For the first type (class 1) distance correlations should be less important than for the second type (class 2) where regulation is mediated (among other processes) by the neighborhood of genes on the genome.

**Figure 6:**
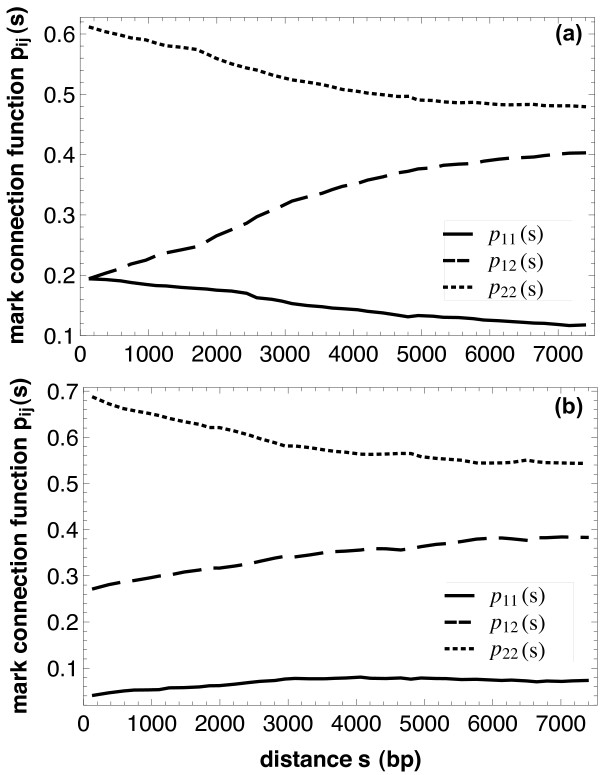
**Mark connection functions**. Mark connection function *p*_*ij*_(*s*) for (a) genes and (b) operons. Analogously as in Figure 5, the full curves denote *p*_11_(*s*), the dotted curves *p*_22_(*s*) and the dashed curves *p*_12_(*s*). The functions show that the maxima in Fig. 5 result only from different frequencies of inter-point distances, while the probabilities of being isolated or regulated depend monotonously on the interpoint distance *s*.

Figure 6 shows the *mark connection functions p*_*ij*_(*s*), again for genes (Figure 6a) and for operons (Figure 6b). All these function are simply monotonous and nearly linear. The curve for *p*_22_(*s*), for example, shows that the probability that two genes (operons) of distance *s *are both isolated is monotonously decreasing. The numerical differences between the values for genes and operons result from the different values of *p*_2_, which are 0.676 and 0.735 for genes and operons, respectively. These functions show that the maxima of the *g*_*ii*_(*s*) result mainly from higher numbers of gene/operon pairs of the corresponding inter-gene/operon distances, while the probabilities that members of such pairs are both monotonously decreasing in *s*. They are decreasing for *i *= 2 for genes and operons, while the function *p*_11_(*s*) is decreasing for genes and increasing for operons. The decrease of *p*_22_(*s*) in both cases (genes and operons) is in good agreement with our expectations that short distances should contribute more strongly in the case of analog control. We believe that the decrease of *p*_11_(*s*) for genes is a consequence of the very strong short-range contributions of intra-operon distances (i.e. of genes within the same operon).

It should be noted that the *partial pair correlation functions g*_*ij*_(*s*) compared to the *mark connection functions p*_*ij*_(*s*) are individually normalized. In contrast to *p*_*ii*_(*s*) we see maxima of *g*_*ii*_(*s*) around 2 kbp. Comparison between the types 1 and 2 shows that regulatory genes are more regularly distributed than isolated genes (as the maximum is higher for *g*_11_(*s*)). We would also like to point out that the estimates of the partial pair correlation function and mark connection function depend continuously on the proportions of class 1 and class 2 genes in this analysis (see also Methods). We thus expect that small fluctuations in the data will leave the main results of our analysis intact.

Both, in the *partial pair correlation functions g*_12_(*s*) and in the *mark connection function p*_12_(*s*) one can see that the two classes (isolated genes and genes involved in regulation) repel each other. On the level of the operons this repulsion is less clearly visible (and has a range up to approximately 2.5 kbp); in general, operons are more irregularly spaced than the genes. In all these cases, this can be explained by the elimination of many short (intra-operon) distances from consideration, when passing from the gene level of description to the operon level.

**Figure 7:**
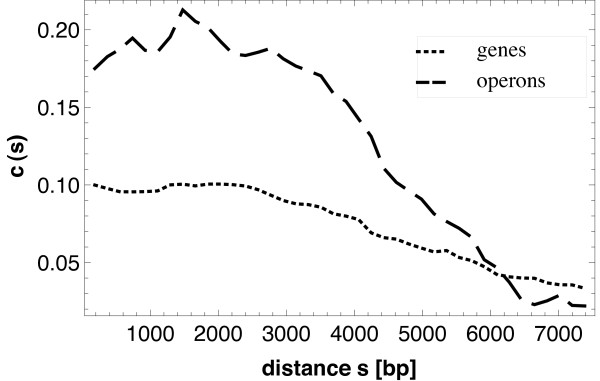
**Connectivity correlation function**. Connectivity correlation function *c*(*s*) for genes (dotted) and operons (dashed). The functions show that the probability that the members of a pair of non-isolated points regulate each other decreases only for distances *s *larger than a value *s*_0 _(approximately 3000 bp for the genes and 2500 for the operons). The weak irregularities of the curve for the operons result from the fact that, while the same estimator is used as for the genes, the number of passive operons is much smaller than that of that of passive genes and so the statistical quality of the results decreases a little.

The second group of correlation functions describes distances between points (genes/operons) under common regulation. First we look at the probability for two regulatory (mark 1) points at a distance *s *that there is a regulation relationship between them. This is expressed in terms of the connectivity correlation function *c*(*s*), which is given in Figure 7. The curves show that there is a critical distance *s*_0 _between 2 and 3 kbp such that for *s *larger than *s*_0 _the probability that the two points regulate another decreases continuously. The numerical values for the operons are clearly larger than those for the genes, indicating a higher systematic (importance of distance for the organization of regulation).

**Figure 8:**
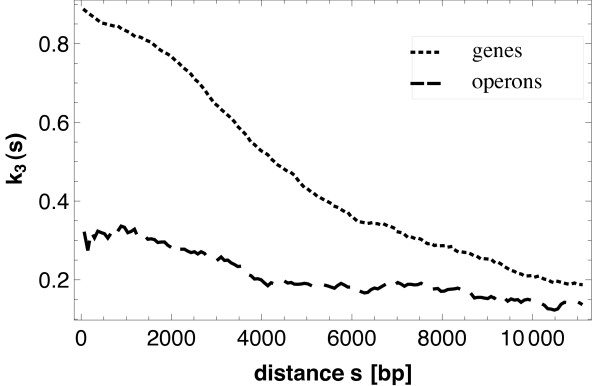
**Control correlation function**. Control correlation function *k*_3_(*s*) for genes (dotted) and operons (dashed). The functions show that the probability that the members of a pair of passive points are regulated by the same point decreases monotonously with increasing distance *s*. As in Figure 7, the weak irregularities of the curve for the operons result from the fact that, while the same estimator is used as for the genes, the number of passive operons is much smaller than that of that of passive genes and so the statistical quality of the results decreases a little.

Finally, Figure 8 shows the curves for the control correlation functions *k*_3_(*s*). Now only passive points are considered, a subset of the regulatory points. The probability of interest is that the two points considered are regulated by the same (active) other point. Since the basic point processes for *c*(*s*) and *k*_3_(*s*) are different, namely 1-points and passive points, no simple inequality between both functions must hold true. The function *k*_3_(*s*) thus indicates that the three objects involved (two regulated genes, one regulator) are preferentially close together.

**Figure 9:**
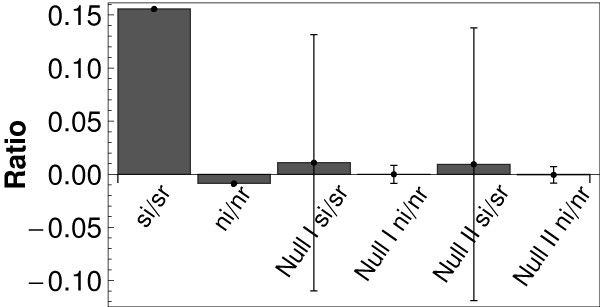
**Comparison with supercoiling-sensitive genes**. Excess of isolated vs. regulatory genes in the supercoiling-sensitive genes, together with a comparison with randomly drawn genes (null model I: randomly selected supercoiling-sensitive genes; null model II: randomly selected isolated genes).

How do the two categories, regulatory genes (class 1) and isolated genes (class 2), compare with experimental information on gene regulation? We used a list of supercoiling-sensitive genes from [21] and compared it with the two categories of genes discussed in our manuscript (*i*: number of isolated genes, and *r*: number of genes involved in regulation). Figure 9 shows the ratio of isolated and regulatory genes for both experimental classes (*s*: number of supercoiling-sensitive genes; *n*: number of non-sensitive genes; *si *then denotes the number of isolated supercoiling-sensitive genes, etc.), normalized by the number of genes in the two categories; for clarity, a value of one (representing equal proportions of genes in both categories) has been subtracted. The first value in Figure 9 is thus ((*si*/*sr*) (*r*/*i*) - 1). If we assume that supercoiling-sensitive genes are genes, for which analog control is systematically more important than digital control, we expect a larger percentage of isolated genes to be in this group. Even though this test can only provide very indirect evidence, the effect is clearly visible in Figure 9, as the first value deviates the strongest from zero. For non-sensitive genes, as well as for all random samplings the values are close to zero. It should be pointed out, however, that the statistical significance is not high enough to form a solid basis for interpretation. The more sophisticated techniques, which lead to the previous figures, are indeed necessary for this.

Our statement that short distances and analog control are qualitatively related can also be checked on the level of this data set. While it should be noted that our key result is a statistical signal emerging from the collective ensemble of genes (and here we show additionally, how these findings can again be cross-validated against high-throughput data), we again resort to the data from [21] and compare a histogram of inter-gene distances obtained from supercoiling-sensitive genes with a histogram obtained from a random selection of genes. The trend towards smaller distances is clearly seen. This figure is included as supplementary information (Additional File 1).

**Figure 10:**
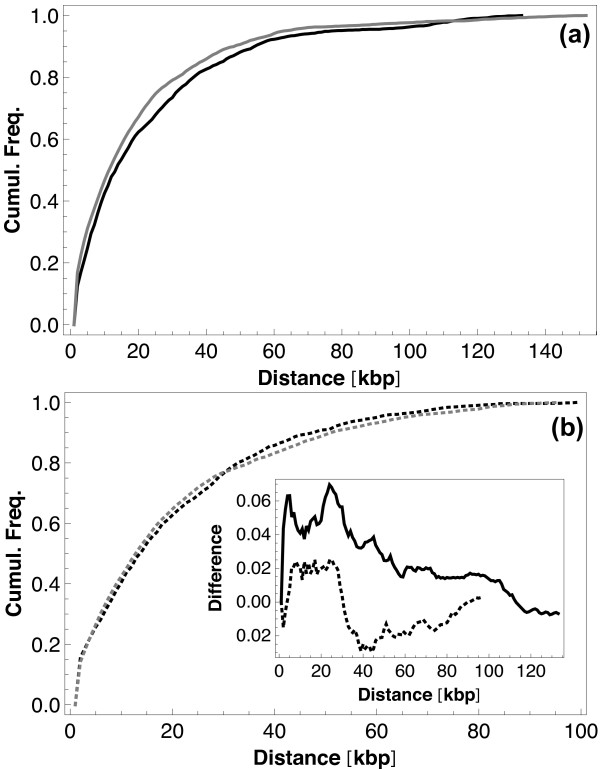
**Comparison with extensive protein occupancy domains (EPODs)**. Cumulative frequencies of distances between genes and extensive protein occupancy domains (EPODs): (a) distances to transcriptionally silenced EPODs (full curves), (b) distances to highly expressed EPODs (dotted curves). In both cases this analysis has been performed independently for the isolated genes (gray curves) and the regulatory genes (black curves). Inset: Differences between the gray and black curves from both parts, i.e. for tsEPODs (full curve in inset) and heEPODs (dotted curve in inset).

The second data set is taken from [22], where the protein occupancy landscape (i.e. the probability per base of a protein binding event) has been measured. The authors distinguish between transcriptionally silenced extensive protein occupancy domains (tsEPOD) and highly expressed extensive protein occupancy domains (heEPOD). Figure 10 shows the distance of isolated genes and regulatory genes from these domains in a cumulative plot. While no substantial difference between the isolated and regulatory genes is seen for the heEPODs (Figure 10b), the distances of isolated genes to tsEPODs are clearly shorter than those of regulatory genes (Figure 10a), pointing again towards a biological significance of this distinction between isolated and regulatory genes and also towards a stronger importance of analog control (mediated by regional binding events of structural proteins to the DNA) for isolated genes.

The inset in Figure 10b summarizes the two parts of Figure 10 by showing the difference between the isolated gene curve and the regulatory gene curve from Figure 10a (full curve in the inset; tsEPODs) and from Figure 10b (dotted curve in the inset; heEPODs), respectively. A particular interesting feature seen in the inset is that at short distances the full curve goes up and the dotted curve goes down, i.e. there are (at short distances) far more isolated genes in the vicinity of transcriptionally silenced EPODs and more regulatory genes in the vicinity of highly expressed EPODs.

**Figure 11:**
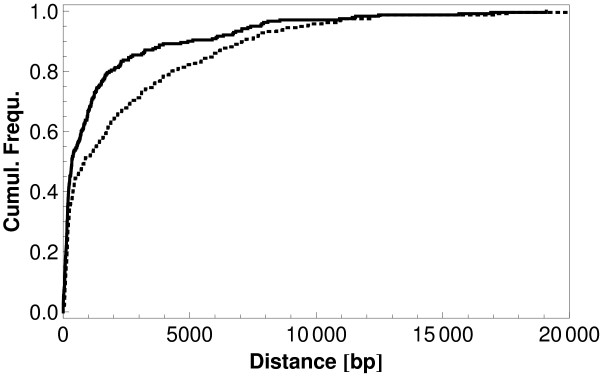
**Distances between regulatory and isolated genes taking orientation into account**. Cumulative frequencies of distances *r *- *i *(full) and *i *- *r *(dashed).

Lastly, we looked at pairs (class 2, class 1) = (*i, r*) of genes, taking into account the orientation of genes on the respective strand. Figure 11 distinguishes between *i *- *r *and *r *- *i *and shows the cumulative distances for these two cases (*r *- *i*: full, *i *- *r*: dotted). We find strong differences between these cases, again suggesting that "isolated" and "regulatory" are meaningful categories for our analysis. Additionally, these differences could indicate that the larger size of the regulatory regions in the *r *category is a contributor to the repulsion we observe between the two categories.

## Conclusions

Patterns (i.e. systematic deviations from randomness in the arrangement of genes) in the genome of *E. coli *have been studied on many different scales.

Here we analyzed another facet of this topic by distinguishing between genes involved and not involved in regulation based on transcription factors. Our key finding is that these two classes, regulatory and isolated genes display a statistical repulsion. Furthermore, the (operon-level) partial pair correlation function has a peak at shorter distances for isolated genes than for regulatory genes. This preference of shorter distances for isolated genes is also visible in the mark connection function and is supportive of our hypothesis that analog control is more important for this class of genes than for the regulatory genes, for which digital control is a longer-ranging alternative.

Whether the statistical properties of inter-gene distances discussed here originate from the need to organize gene regulation or from the dynamics of genome rearrangement cannot be ultimately decided based on the data at hand.

Minimal models of genome arrangement dynamics and its impact on gene expression could be a useful tool for deciding whether the distance pattern between genes is indirectly shaped (and therefore deviates from pure randomness) by these dynamics, rather than being evolutionarily constraint to contribute more directly to gene regulation.

The statistical differences between isolated and regulatory genes described here suggest that, indeed, the genes currently classified as isolated from the perspective of the available TRN are systematically different from the genes involved in regulation. We by no means want to suggest that (a) these genes are indeed unregulated nor (b) that the current version of RegulonDB (version 6.2) is complete. However, when considering the extreme cases of isolated genes being just gaps in the database and, on the other hand, isolated genes being systematically regulated by other means, our results support the latter view.

Even though we consider our findings in an evolutionary context (by making visible some deviations from randomness of the gene distances in the *E. coli *genome, which can only be understood evolutionarily) we here do not directly discuss the comparative genomics aspect of it. It would be particularly interesting to analyze the degree of evolutionary conservation as a function of the distance between genes and separately for the two categories of genes. A hypothesis for such an extension of our analysis could be that pairs of genes contributing strongly to the patterns we observe, have a higher degree of evolutionary conservation. This is, indeed, a whole work package we plan to tackle in a future investigation.

Eventually one needs to arrive at a more holistic view of the system and explain the interplay between gene arrangement, DNA binding site distributions, physical properties of DNA binding sites, the architectural properties of the transcriptional regulatory network and the spatial gene expression patterns, in order to understand the binding site code behind global gene expression and to unravel the universal design principles of transcriptional regulation.

## Methods

### Point process statistics

In the statistical analyses of this paper, the genes are considered as points on a circle *C*, the circular chromosome of *E. coli*. Thus, a random system of points is analysed, which leads to the application of methods of point process statistics. (The term "process" is related to early applications where the points were time instants. Also the term "stationary" is related to these applications; "homogeneous" could be an equivalent.) These methods have been mainly developed for the planar (*d *= 2) and spatial (*d *= 3) case, but can be easily applied also in the one-dimensional (*d *= 1) case considered here. So our main reference is Illian et al. (2008) [23].

Similarly to the investigations of [5,6] we assume that the point pattern belongs to a "stationary" point process, i. e. that the point distribution is rotation invariant. This implies that the local point density does show only irregular fluctuations, as it is the case. Thus it makes sense to speak about the "intensity", the mean number of points per length unit. As in [23] it is denoted here by *λ*.

The points considered are marked. There are two marks, namely "1" and "2", where "1" stands for "regulatory" and "2" for "isolated". The fraction of *i*-points is denoted by *p*_*i*_, for *i *= 1, 2. Note that *p*_*i *_can be interpreted as the probability that a randomly chosen point has the mark *i*. Furthermore, the probabilities *p*_1 _and *p*_2 _make sense, where *p*_*i *_is the fraction of *i*-points (a point with mark *i*) in the point process. It can be interpreted as the probability that a randomly chosen point is an *i*-point.

The statistical analysis uses a series of summary characteristics, which have been successfully employed in spatial point process statistics. All these functions depend on a variable *s*, which is a distance. In all cases this is the shortest distance along the circular genome.

All these function can be called "correlation functions", but not all include only point pairs; therefore some of them are not second-order characteristics.

The best known function is the *pair correlation function g*(*s*), which is explained here as in [23], p. 219, since the explanation there is closer to the "two-point interpretation" used for explaining the other functions. (The explanation in Warren and ten Wolde is different but equivalent.)

Consider two points *x *and *y *on *C *of distance *s *and two infinitesimal length elements of lengths *dx *and *dy *centred at *x *and *y*. Denote the probability that in the two elements there is each a point by *p*(*x, y*). This probability is given by the so-called product density *ϱ*(*x, y*) as *p*(*x, y*) = *ϱ*(*x, y*)*dxdy*.

In the stationary case (which is assumed), *ϱ*(*x, y*) depends only on the distance *s *of *x *and *y*, and the simpler symbol *ϱ*(*s*) is used. The pair correlation function is then *g*(*s*) = *ϱ*(*s*)/*λ*^2^.

The normalisation by division by *λ*^2 ^makes that for large *r*, *g*(*r*) approximates 1. Values of *g*(*r*) larger than 1 for small *r *indicate clustering, while values smaller than 1 indicate some tendency of regularity or repulsion between the points. See the discussion of the information given by a pair correlation function in [23], pp.219.

The *partial pair correlation functions g*_*ij*_(*s*) are defined using refined product densities *ϱ*(*s*) where one of the points in the infinitesimal intervals is an *i*-point and the other a *j*-point, see [23], p. 325. These functions are normalized by *p*_*i*_*p*_*j *_*λ *^2^, which leads to *g*_11_(*s*), *g*_12_(*s*) and *g*_22_(*s*). The *g*_*α*_(*s*) in [5] are similar to *g*_11_(*s*).

Again, the normalisation leads to values around 1 for large *s*, and also the general interpretation is similar to that of *g*(*r*), see [23], pp.325. For *i *≠ *j *the relations between different sorts of points are characterized.

For example, values smaller than 1 for *g*_*ij*_(*r*) indicate some tendency of repulsion or inhibition between points of the different types *i *and *j*.

The *mark connection functions p*_*ij*_(*s*) are defined by(1)

of course only for such *s *where the denominator is positive, see [23], p. 331. It can be interpreted as the conditional probability that two points at distance *s *have marks *i *and *j*, given that these points are in the point process. These probabilities have the following behavior for large *s*:(2)

and(3)

for *i *≠ *j*. It is useful to consider the *mark connection functions *additionally to the partial pair correlation functions since they characterize the occurrence of the point types with eliminated influence of fluctuations in point density; see [23], p. 332.

Comparison of the Figures 5 and 6 shows the power of this approach. The curves in Figure 5 are heavily dominated by the frequencies of point distances, which show for the genes a maximum at around *s *= 2000...3000, while Figure 6 shows the true nature of the marking: the probability that two points of distance *s *have, for example, both mark 2 decreases monotonically with *s*.

The connectivity correlation function *c*(*s*) is also a characteristic of a conditional nature. It is defined by(4)

where *ϱ*_*conn*_(*s*) is a quantity which yields the probability that between the points *x *and *y *in the infinitesimal intervals above, if both are regulatory (both have mark 1), there is a direct regulatory relationship, i. e. one of them regulates the other or both regulate the other. It is similar to the connectivity function in [23], p. 249, and can be interpreted as the conditional probability that between two regulatory points at distance *s *there is a direct regulatory relationship.

Finally, the control correlation function *k*_3_(*s*) is defined by(5)

It is defined for the sub-point process of all points that are regulated by other points ("passive" points, a subset of all 1-points); its product density is denoted by *ϱ*_*pp*_(*s*). Furthermore, *ϱ*_3_(*s*) is a quantity which yields the probability that for two passive points *x *and *y *in the infinitesimal intervals above there exists a third point which regulates both *x *and *y*. Thus, *k*_3_(*s*) can be interpreted as the conditional probability that for two passive points at distance *s *there is a third point which controls both of them.

### Transcriptional regulatory network and spatial distribution of genes

We obtained the data from RegulonDB (version 6.2) [10], which is a database specifically dedicated to the transcriptional regulation of *E. coli*. A total number of 4548 genes are included in this database, of which 1474 bear information about their transcriptional regulation and thus have been classified as class 2 genes.

## Authors' contributions

DS and MH conceived the study. HS, DS and NS analyzed the data. DS, HS, NS and MH wrote the manuscript. All authors read and approved the final manuscript.

## Supplementary Material

Additional file 1 **Distances among supercoiling-sensitive genes and other genes**. Histogram of distances observed between supercoiling-sensitive genes (dark gray) and a random sample of other genes (light gray). The inset shows the corresponding cumulative distance plot. Click here for file
