# Supplementary figures and images for: Ranges of control in the transcriptional regulation of Escherichia coli
Source: BMC Syst Biol. 2009 Dec 24;3:119. doi: 10.1186/1752-0509-3-119 (PMC2804738; doi:10.1186/1752-0509-3-119)

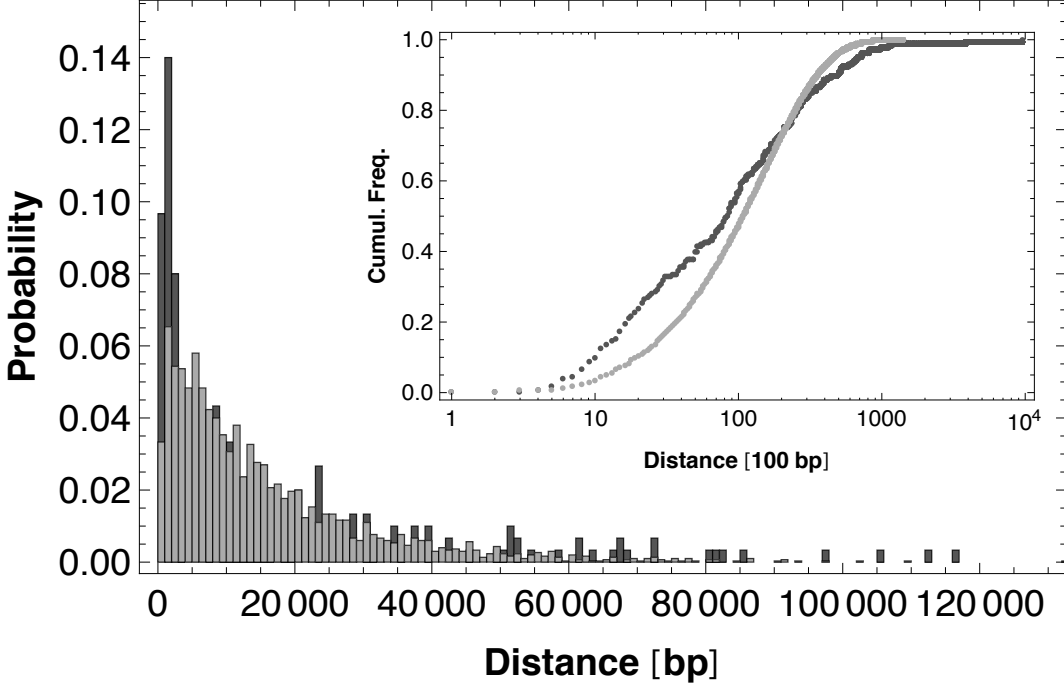

Supplement: Additional file 1 — Distances among supercoiling-sensitive genes and other genes. Histogram of distances observed between supercoiling-sensitive genes (dark gray) and a random sample of other genes (light gray). The inset shows the corresponding cumulative distance plot. [file 1752-0509-3-119-S1.PDF]
